# Association between red blood cell distribution width to albumin ratio and periodontitis in US adults: A cross-sectional study

**DOI:** 10.1097/MD.0000000000050414

**Published:** 2026-08-28

**Authors:** Lina Zhu, Ke Yang, Qian Zheng, Wenhao Qian

**Affiliations:** a Laboratory of Dental Biomaterials and Tissue Regeneration, Shanghai Xuhui District Stomatological Hospital, Shanghai, PR China; b College of Mechanical and Electrical Engineering, China JiLiang University, Hangzhou, PR China; c Department of Oral Medicine, Hangzhou Red Cross Hospital, Hangzhou, PR China.

**Keywords:** albumin (ALB), National Health and Nutrition Examination Survey (NHANES), periodontitis, red blood cell distribution width (RDW), red blood cell distribution width to albumin ratio (RAR)

## Abstract

Research on the potential relationship between RAR and periodontitis remains unclear. This study examines the association between RAR and periodontitis among US adults, utilizing data from the National Health and Nutrition Examination Survey (NHANES; 2009–2014). Weighted multivariate logistic regression analyses, restricted cubic spline curve (RCS) models, and subgroup analyses were utilized to evaluate the relationship between RAR and periodontitis. Additionally, sensitivity analysis was used to evaluate the robustness of the results. This study included 8529 participants, 3928 (46.05%) with moderate or severe periodontitis. After full adjustment for potential confounders, RAR was significantly associated with periodontitis (OR = 1.23, 95% CI: 1.02–1.49). When RAR were categorized as 4 groups, periodontitis were possibly associated with Q2 (OR = 1.02, 95% CI: 0.86–1.21), Q3 (OR = 1.31, 95% CI: 1.09–1.58), Q4 (OR = 1.21, 95% CI: 0.95–1.54). The RCS models indicated a nonlinear association between RAR and periodontitis (*P* < .001). Additionally, subgroup analyses and sensitivity analyses validated the robustness of the finding regarding the association. This study suggests that higher RAR level was associated with higher odds of periodontitis.

## 1. Introduction

Periodontitis is a chronic inflammatory disease caused by plaque biofilm, which affects all periodontal supporting tissues, such as gingiva, periodontal ligament, cementum, and alveolar bone.^[1,2]^ It is the main cause of tooth loss in adults globally, and the World Health Organization has classified severe periodontitis as the sixth most prevalent disease in the world. The direct healthcare costs and indirect socioeconomic losses make periodontitis an important public health burden.^[3]^ Periodontitis, as a common chronic inflammatory disease, is closely associated with a variety of systemic diseases, and its pathomechanism involves an aberrant host immune-inflammatory response to microorganisms accompanied by significant oxidative stress.^[4,5]^ Therefore, understanding the association between systemic inflammatory states and periodontitis is imperative for enhancing periodontal health.

The red blood cell distribution width (RDW), a routine blood parameter reflecting the heterogeneity of erythrocyte volume (unequal sizes), has attracted attention for its association with chronic inflammation and oxidative stress.^[6,7]^ Studies have demonstrated that RDW levels are significantly higher in patients with chronic periodontitis than in periodontally healthy individuals, suggesting that it may serve as a simple and cost-effective indicator of the chronic inflammatory state of periodontal tissues.^[8,9]^ Meanwhile, serum albumin (ALB), an important negative acute temporal response protein, has anti-inflammatory and antioxidant properties.^[10]^ Hypoalbuminemia is associated with malnutrition and a systemic inflammatory state and has been shown to correlate negatively with the severity of periodontal disease in the elderly population and in some patients.^[11,12]^

The red blood cell distribution width to albumin ratio (RAR) has been proposed as a composite blood parameter of inflammation and nutritional status. Recent studies have shown that it is associated with a variety of diseases linked to systemic inflammation, such as cardiovascular disease,^[13,14]^ sepsis,^[15]^ and acute kidney injury.^[16]^ However, although the independent associations of RDW and periodontitis, and albumin and periodontitis have been preliminarily explored, systematic investigation into the relation of RAR and periodontitis remains unexplored.

This study aimed to explore the potential association between RAR and periodontitis by using cross-sectional analysis of NHANES data from 2009–2014.

## 2. Materials and methods

### 2.1. Study population

The NHANES (https://www.cdc.gov/nchs/NHANES/) is a nationally representative, cross-sectional study conducted by the National Center for Health Statistics to assess the health and nutritional status of US adults and children,^[17]^ which collected a large amount of data, including demographics, dietary intake, physical examinations, and laboratory tests. Written informed consent was provided by all participants. This study followed the guidelines of the Strengthening the Reporting of Observational Studies in Epidemiology (STROBE). The data selected for this study were from NHANES 2009–2014.

**Figure 1.**
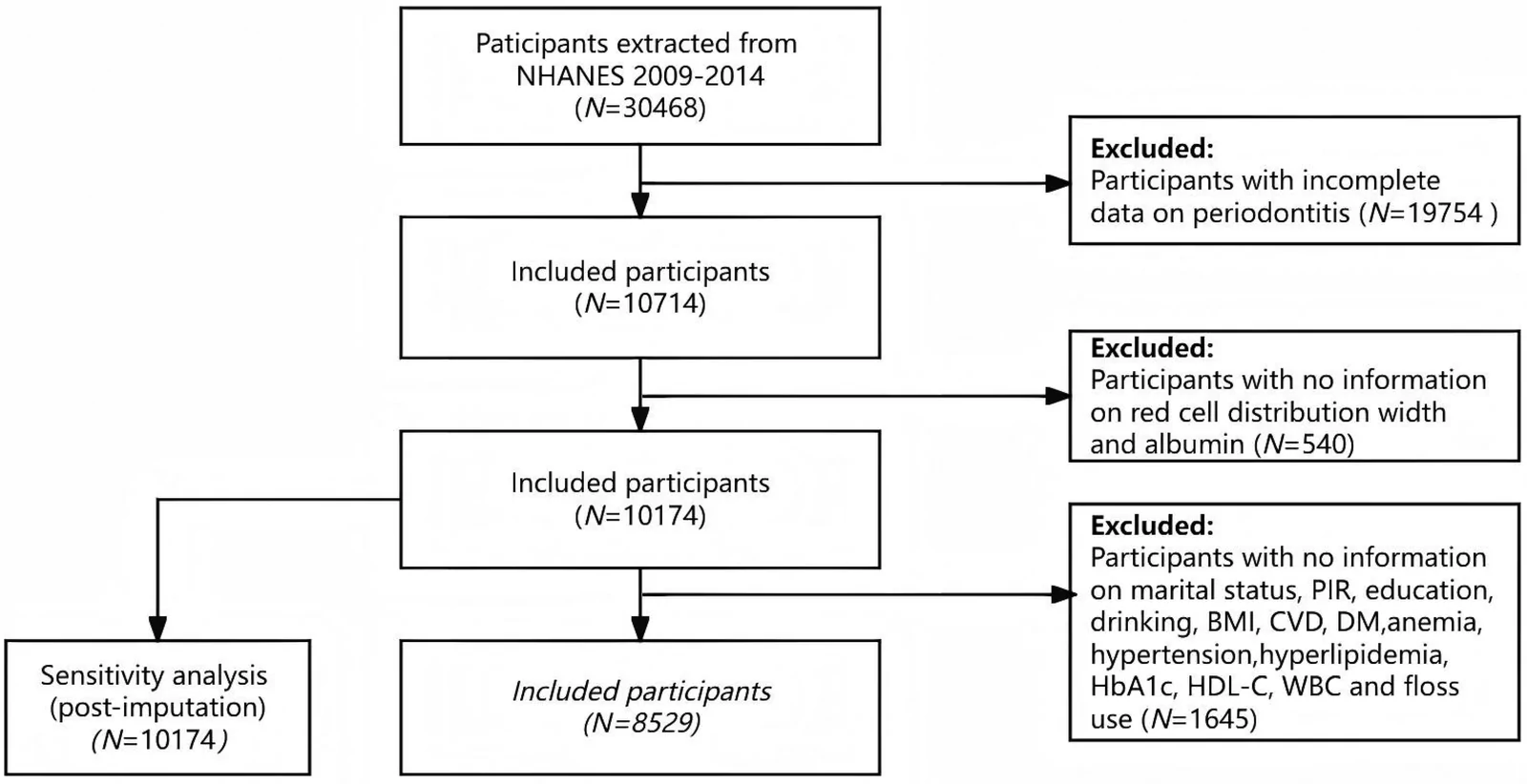
Participant inclusion flowchart. BMI = body mass index, CVD = cardiovascular disease, DM = diabetes mellitus, HbA1c = glycosylated hemoglobin level, HDL-C = high-density lipoprotein cholesterol, NHANES = the National Health and Nutrition Examination Survey, PIR = poverty income ratio, RDW = red cell distribution width, WBC, white blood cell.

A total of 30,468 participants were initially collected. Those with missing data on periodontitis (N = 19,754), RDW and ALB (N = 540), and marital status, poverty-to-income ratio (PIR), education, drinking status, body mass index (BMI), cardiovascular disease (CVD), diabetes mellitus (DM), hypertension, hyperlipidemia, anemia, glycosylated hemoglobin level (HbA1c), high-density lipoprotein cholesterol (HDL-C), white blood cell (WBC), and floss use (N = 16,248) were excluded. Finally, a total of 8529 participants were enrolled, as shown in Figure 1.

### 2.2. Definition of periodontitis

Periodontal data were derived from Oral Health-Periodontal in Examination Data of NHANES 2009–2014.^[17]^ A comprehensive dental examination (excluding third molars) was performed by a certified dentist. Six sites were measured per tooth. Periodontitis classification utilized measurements of clinical attachment loss (CAL) and periodontal pocket depth (PPD) recorded at four interproximal sites per tooth, according to the following criteria.^[18]^ The degree of periodontitis is defined according to the criteria of the Centers for Disease Control and Prevention and the American Academy of Periodontology (CDC/AAP).^[19]^ Specifically, severe periodontitis was defined as the presence of ≥2 interproximal sites with CAL ≥ 6 mm (nonadjacent teeth) and ≥1 interproximal site with a PPD of ≥5 mm; moderate periodontitis required either ≥2 interproximal sites with a CAL of ≥4 mm (nonadjacent teeth) or ≥2 interproximal sites with a PPD of ≥5 mm (non-adjacent teeth). Participants who did not meet these thresholds were categorized as having no/mild periodontitis. Participants were categorized into 2 groups: a no/mild periodontitis group and a moderate/severe periodontitis group. This categorization has been widely used in previous studies related to periodontitis and systemic health.^[20-22]^

### 2.3. Measurement of RAR

Blood samples were collected at the NHANES Mobility Examination Center (MEC). RDW was measured as part of the complete blood count using the BeckmanCoulter MAXM instrument, which uses the Beckman Coulter method for counting, screening, automated dilution, and mixing to process the samples.^[7]^ ALB level was measured using the DcX800 method, which utilizes a bichromatic digital endpoint method to determine ALB concentration. In this process, ALB binds to bromocresol purple reagent to form a complex. The system monitors the change in absorbance at 600 nm, which is proportional to the concentration of albumin in the sample.^[23]^ RAR was calculated from RDW/ALB. In order to explore the exact relationship between RAR and the odds of periodontitis, we used RAR as a continuous variable and 4 categorical variables.

### 2.4. Covariates

The potential covariates were selected according to the literature^[24–26]^: age (year), sex (male/female), race (non-Hispanic White, non-Hispanic Black, Mexican American, others), education (less than ninth grade, high school or equivalent and above), marital status (married/living with partner, never married/other), poverty-to-income ratio (PIR: ≤1.30, 1.31–3.50, >3.50), BMI, hypertension (yes/no), DM (yes/no), hyperlipidemia (yes/no), CVD (no/yes), anemia (no/yes), HbA1c, HDL-C, WBC, drinking status, and floss use. Drinking status was divided into 3 groups: never (<12 drinks in life), former (did not drink last year but drank more than 12 drinks in their lifetime), and current (≥12 drinks in any 1 year and did drink last year). Floss use is divided into 4 groups: never (0 times per week), rarely (1–2 d/wk), moderately (3–5 d/wk), and frequently (6–7 d/wk). The diagnosis of DM was established using various criteria including self-reported physician diagnosis, a fasting blood glucose level ≥ 7.0 mmol/L, a glycosylated hemoglobin level (HbA1c) ≥ 6.5%, or current use of hypoglycemic drugs. Similarly, hypertension diagnosis was determined based on self-reported physician diagnosis, a diastolic blood pressure measurement of ≥80 mm Hg, a systolic blood pressure reading of ≥130 mm Hg, or current use of antihypertensive medications. Anemia was defined with a cutoff of 12g/dL for females and 13 g/dL for males. Hyperlipidemia was identified if participants met any of the following criteria: elevated low-density lipoprotein cholesterol (LDL-C) (≥130 mg/dL), total cholesterol (≥200 mg/dL), or triglycerides (≥150 mg/dL); reduced HDL-C (<40 mg/dL in men or <50 mg/dL in women); or reported use of lipid-lowering medications. Physical activity (PA) was divided into 3 groups: inactive (PA = 0), insufficiently active (0 < PA < 600 metabolic equivalent (MET)-min/wk), and active (PA ≥ 600 MET-min/wk), respectively.

### 2.5. Statistical analysis

According to NHANES analytic guidelines, complex sampling design and sampling weights were incorporated into our analyses. This study employed 3 weighting variables from the NHANES database – SDMVSTRA, SDMVPSU, and WTMEC2YR. The sampling weight was calculated using the following formula: WTMEC2YR/3. Continuous variables are presented as mean and standard error (SE), with intergroup comparisons conducted using *t* tests. Categorical variables are presented as unweighted counts and weighted proportions [n (%)], with intergroup comparisons performed using chi-square tests or Fisher exact probability test. Confounding factors was screened by weighted univariate logistic regression analysis. Weighted multivariate logistic regression analyses were performed to assess the association between RAR and periodontitis. Three models were used: Model 1 was an unadjusted crude model. Model 2 adjusted for age, sex, race, education level, marital status, PIR, and BMI. Model 3 adjusted for the covariates in model 2 plus hypertension, DM, hyperlipidemia, CVD, anemia, HbA1c, HDL-C, WBC, drinking status, physical activity, and floss use. *P* values < .05 were considered statistically significant. To further assess the dose-response associations between the RAR and periodontitis, restricted cubic spline curve (RCS) analysis was performed using 4 nodes at the fifth, 35th, 65th, and 95th percentiles of the RAR. In addition, subgroup analyses were conducted to test the robustness of the results, stratified by age, sex, DM, CVD, and anemia. Finally, to ensure the robustness of the research findings, we conducted a sensitivity analysis. To assess the impact of missing data on the results, sensitivity analyses were performed by multiple imputation by chained equation (MICE) for predictive mean matching, generating 5 imputed datasets. Statistical analyses were carried out using R software (v4.2.2; R Foundation for Statistical Computing; http://www.R-project.org) and Free Statistics analysis platform (v2.2.0, Beijing Free Clinical Medical Technology Co., Ltd.). Descriptive statistics were calculated for all participants, and statistical significance was assessed using a 2-tailed test with a *P*-value threshold of < 0.05.

### 2.6. Ethics approval and consent to participate

This study followed the ethical standards of the Helsinki Declaration and its amendments or equivalent standards. The data used were obtained from the NHANES survey approved by the NCHS Ethics Review Board. All participants in the study provided informed consent prior to enrollment. As this study involved secondary analysis of the NHANES database and met the requirements of Strengthening the Reporting of Observational Studies (STROBE) in Epidemiology, no additional ethical approval was required. For more information on NHANES methodology and ethical standards, please visit the Centers for Disease Control and Prevention (CDC) and NCHS official websites: https://www.cdc.gov/nchs/nhanes/irba98.htm.

## 3. Results

### 3.1. Baseline characteristics of the participants

This study included 8529 participants, with weighted results extrapolated to represent the demographic characteristics of 12,02,20,884 adults in the United States, 50.70% of whom were male and 49.30% female. The weighted mean age was 51.14 years (SE = 0.25). Among these participants, 5547 (69.73%) were married and 2982 (30.27%) were unmarried. The study also found that 3928 participants (46.05%) had periodontitis. Table 1 shows comparisons of baseline information between the periodontitis and non-periodontitis groups.

**Table 1:** Baseline characteristics of participants stratifed by periodontitis status.

Variables	Total	Non-periodontitis/mild periodontitis	Moderate/severe periodontitis	*P*-value
Unweighted population, *N*	N = 8529	N = 4601	N = 3928	
Weighted population,*N*	N = 12,02,20,884	N = 7,51,64,819	N = 4,50,56,064	
Age, mean (SE), yr	51.14 (0.25)	48.44 (0.29)	55.66 (0.37)	<.001
Sex, no. (%)				<.001
Male	4340 (50.70%)	1987 (45.19%)	2353 (59.90%)	
Female	4189 (49.30%)	2614 (54.81%)	1575(40.10%)	
Race, no. (%)				<.001
Non-Hispanic White	3939 (71.48%)	2405 (76.28%)	1533 (63.48%)	
Non-Hispanic Black	1698 (9.78%)	736 (7.59%)	962 (13.43%)	
Mexican American	1157 (7.45%)	499 (5.64%)	658 (10.45%)	
Others	1735 (11.29%)	961 (10.49%)	774 (12.64%)	
Education, no. (%)				<.001
Less than ninth grade	712 (4.31%)	219 (2.30%)	493 (7.67%)	
High school or equivalent	2976 (30.47%)	1263 (23.95%)	1713 (41.35%)	
Above high school	4841 (65.22%)	3119 (73.75%)	1722 (50.98%)	
Marry, no. (%)				<.001
Married/living with partner	5547 (69.73%)	3127 (73.22%)	2420 (63.90%)	
Never married/other	2982 (30.27%)	1474 (26.78%)	1508 (36.10%)	
PIR, no. (%)				<.001
≤1.30	2477 (18.10%)	1020 (13.20%)	1457 (26.27%)	
1.31–3.50	3037 (34.26%)	1540 (30.75%)	1497 (40.12%)	
>3.50	3015 (47.64%)	2041 (56.05%)	974 (33.61%)	
Drinking status, no. (%)				<.001
Never	2210 (19.92%)	1146 (19.08%)	1064 (21.33%)	
Former	944 (9.61%)	371 (7.26%)	573 (13.54%)	
Current	5375 (70.47%)	3084 (73.67%)	2291 (65.14%)	
Physical activity, no. (%)				<.001
Inactive	2070 (21.25%)	987 (19.08%)	1083 (24.87%)	
Insufficiently active	1264 (14.42%)	716 (15.49%)	548 (12.64%)	
Active	5195 (64.33%)	2898 (65.43%)	2297 (62.50%)	
BMI, mean (SE), kg/m^2^	29.25 (0.12)	29.09 (0.14)	29.53 (0.17)	.01
CVD, n (%)				<.001
No	7779 (92.45%)	4345 (95.02%)	3434 (88.16%)	
Yes	750 (7.55%)	256 (4.98%)	494 (11.84%)	
Hyperlipidemia, no. (%)				.01
No	2093 (24.43%)	1204 (25.58%)	889 (22.50%)	
Yes	6436 (75.57%)	3397 (74.42%)	3039 (77.50%)	
Hypertension, no.(%)				<.001
No	4718 (59.11%)	2879 (64.51%)	1839 (50.11%)	
Yes	3811 (40.89%)	1722 (35.49%)	2088 (49.89%)	
DM, no. (%)				<.001
No	6910 (85.36%)	3977 (89.18%)	2933 (78.99%)	
Yes	1619 (14.64%)	624 (10.82%)	995 (21.01%)	
Floss use, no. (%)				<.001
Never	2668 (27.15%)	1152 (23.04%)	1516 (34.00%)	
Rarely	1404 (17.97%)	852 (19.48%)	552 (15.45%)	
Moderately	1539 (20.51%)	990 (23.40%)	549 (15.69%)	
Frequently	2918 (34.37%)	1607 (34.08%)	1311 (34.86%)	
HbA1c, mean (SE), g/dL	5.69 (0.02)	5.56 (0.02)	5.90 (0.03)	<.001
HDL-C, mean (SE), mmol/L	1.38 (0.01)	1.40 (0.01)	1.34 (0.01)	<.001
WBC, mean (SE), 1000 cells/uL	7.07 (0.04)	6.91 (0.04)	7.33 (0.05)	<.001
Anemia, no.(%)				.05
No	7725 (93.04%)	4225 (93.53%)	3500 (92.22%)	
Yes	804 (6.96%)	376 (6.47%)	428 (7.78%)	
Albumin, mean (SE), g/dL	4.28 (0.01)	4.30 (0.01)	4.24 (0.01)	<.001
RDW, mean (SE), %	13.12 (0.02)	13.06 (0.03)	13.22 (0.03)	<.001
RAR, mean (SE), %/g/dL	3.09 (0.01)	3.06 (0.01)	3.14 (0.01)	<.001
RAR, no. (%)				<.001
Q1 (<2.86)	2122 (28.91%)	1314 (32.41%)	808 (23.09%)	
Q2 (2.86–3.05)	2140 (26.85%)	1218 (28.14%)	922 (24.69%)	
Q3 (3.05–3.33)	2130 (23.54%)	1062 (21.14%)	1068 (27.56%)	
Q4 (≥3.33)	2137 (20.69%)	1007 (18.31%)	1130 (24.67%)	

BMI = body mass index, CVD = cardiovascular disease, DM = diabetes mellitus, HbA1c = glycosylated hemoglobin level, HDL-C = high-density lipoprotein cholesterol, PIR = poverty–income ratio, RAR = the red blood cell distribution width to albumin ratio, RDW = red blood cell distribution width, SE = standard error, WBC = white blood cell.

### 3.2. Association between RAR and periodontitis

Based on univariable logistic regression analysis, age, sex, race, marital status, PIR, education level, drinking status, hypertension, DM, hyperlipidemia, CVD, anemia, floss use, physical activity, HbA1c, HDL-C, and WBC (except BMI) were significantly related to periodontitis (all *P* < .001; Table S1, Supplemental Digital Content 1).

Higher RAR levels were associated with a higher odds of periodontitis (Table 2). When RAR was assessed as a continuous variable, model 1 (unadjusted for covariates) showed a significant association between RAR and periodontitis (OR = 1.62; 95% CI: 1.42–1.84). In model 3, after adjusting for all covariates, there was a positive association between RAR and periodontitis (OR = 1.23; 95% CI: 1.02–1.49). The association remained significant after changing RAR from a continuous to a categorical variable. There was a higher risk of periodontitis in the highest group (Q4) by 21% compared to the lowest group (Q1) after adjusting all covariates (OR = 1.21; 95% CI: 0.95–1.54). The risk of periodontitis was elevated by 4%, 31%, and 21% in Q2, Q3, and Q4, respectively, and the trend test (*P = *.02) indicates that the results are relatively reliable.

**Table 2:** Multivariable regression analysis of the association between the red blood cell distribution width to albumin ratio and periododntitis.

Variable	Model 1 OR (95% CI)	*P*-value	Model 2 OR (95% CI)	*P*-value	Model 3 OR (95% CI)	*P*-value
RAR	1.61 (1.42–1.84)	<.001	1.26 (1.06–1.49)	.01	1.23 (1.02–1.49)	.04
RAR (category)						
Q1 (<2.86)	Ref.		Ref.		Ref.	
Q2 (2.86–3.05)	1.23 (1.06–1.43)	.01	1.04 (0.89–1.23)	.59	1.02 (0.86–1.21)	.77
Q3 (3.05–3.33)	1.83 (1.57–2.13)	<.001	1.36 (1.13–1.64)	.002	1.31 (1.09–1.58)	.01
Q4 (≥3.33)	1.89 (1.60–2.23)	<.001	1.30 (1.04–1.63)	.02	1.21 (0.95–1.54)	.12
*P* for trend		<.001		.003		.02

Model 1: no adjustment was made for any covariates.

Model 2: adjusted for age, sex, race,education,marital status, PIR, BMI.

Model 3: adjusted for the covariates in model 2 plus hypertension, DM, hyperlipidemia, CVD, anemia, HbA1c, HDL-C, WBC, drinking status, physical activity, floss use.

BMI = body mass index, CI = confidence interval, CVD = cardiovascular Disease, DM = diabetes mellitus, HbA1c = glycosylated hemoglobin level, HDL-C = high-density lipoprotein cholesterol, OR = odds ratio, PIR = poverty income ratio, RAR = the red blood cell distribution width to albumin ratio, Ref. = reference, WBC = white blood cell.

**Figure 2.**
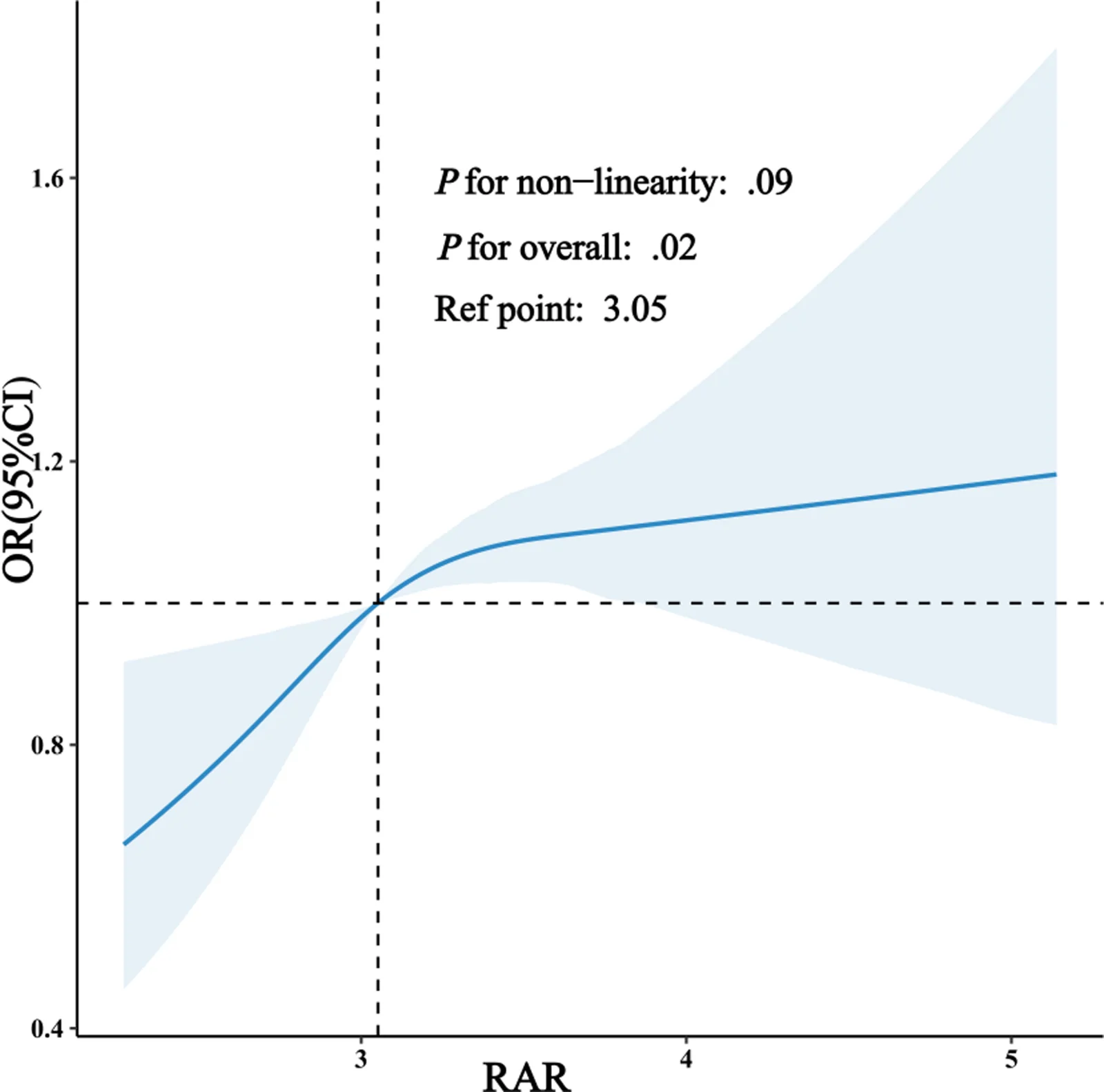
Restricted cubic spline fitting for the association between the red blood cell distribution width to albumin ratio and periodontitis. Adjusted for age, sex, race,education,marital status, PIR, BMI, hypertension, DM, hyperlipidemia, CVD, anemia, HbA1c, HDL-C, WBC, drinking status, physical activity, floss use. BMI = body mass index; CI = confidence interval, CVD = cardiovascular disease, DM = diabetes mellitus, HbA1c = glycosylated hemoglobin level, HDL-C = high-density lipoprotein cholesterol, OR = odds ratio, PIR = poverty income ratio, Ref. = reference, WBC = white blood cell.

Figure 2 presents a linear association between RAR and periodontitis (*P* for nonlinearity = .09). The solid line indicates the estimated odds of periodontitis, and the dashed lines represent point-wise 95% confidence intervals. Only 99.5% of the data is shown.

### 3.3. Subgroup analysis

**Figure 3.**
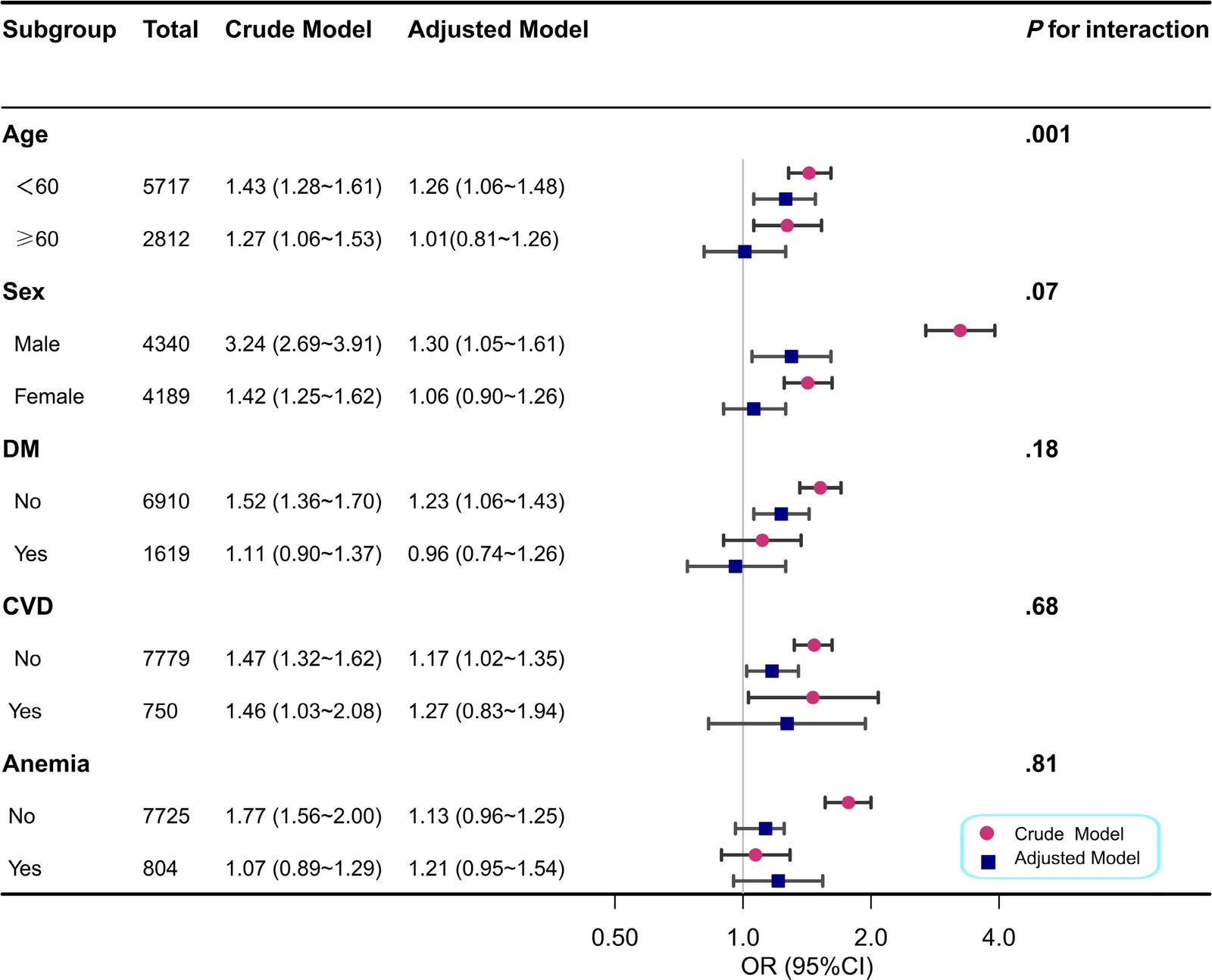
Association between the red blood cell distribution width to albumin ratio and periodontitis in different subgroups. Adjusted for age, sex, race,education,marital status, PIR, BMI, hypertension, DM, hyperlipidemia, CVD, anemia, HbA1c, HDL-C, WBC, drinking status, physical activity, floss use. BMI = body mass index; CI = confidence interval, CVD = cardiovascular disease, DM = diabetes mellitus, HbA1c = glycosylated hemoglobin level, HDL-C = high-density lipoprotein cholesterol, OR = odds ratio, PIR = poverty income ratio, Ref. = reference, WBC = white blood cell.

Subgroup analysis was used to assess the relationship of RAR with periodontitis in subgroups stratified according to age, sex, DM, CVD, and anemia (Fig. 3). The findings showed that age played a significant role in influencing this association between RAR and periodontitis (*P* for interaction < .001). Specifically, among individuals younger than 60 years old, a significant positive association between RAR and periodontitis was observed. Conversely, in the group of individuals older than 60 years old, this association was not observed. None of the other subgroups demonstrated significant effects on the association between RAR and periodontitis.

### 3.4. Sensitivity analysis

To validate result stability, a sensitivity analysis was executed, and based on complete data with imputed variables from multiple imputation, the relationship between RAR and periodontitis remained reliable. In model 3, after controlling for all covariates, there was a positive relationship between RAR and periodontitis when assessed as continuous variables (OR = 1.14; 95% CI: 0.95–1.37). When assessed as a categorical variable, compared to the Q1 group, the adjusted OR values for Q2, Q3, and Q4 were 1.02 (95% CI: 0.87–1.18), 1.22 (95% CI: 1.01–1.46), and 1.13 (95% CI: 0.90–1.42), respectively (Table 3).

**Table 3:** Sensitivity analysis after multiple imputation of missing data.

Variable	Model 1 OR (95% CI)	*P*-value	Model 2 OR (95% CI)	*P*-value	Model 3 OR (95% CI)	*P*-value
RAR	1.49 (1.33–1.66)	<.001	1.18 (1.01–1.36)	.03	1.14 (0.95–1.37)	.14
RAR (category)						
Q1 (<2.86)	Ref.		Ref.		Ref.	
Q2 (2.86–3.05)	1.26 (1.10–1.44)	.001	1.04 (0.90–1.20)	.62	1.02 (0.87–1.18)	.80
Q3 (3.05–3.33)	1.75 (1.50–2.04)	<.001	1.28 (1.07–1.53)	.01	1.22 (1.01–1.46)	.04
Q4 (≥3.33)	1.78 (1.53–2.07)	<.001	1.22 (0.99–1.50)	.06	1.13 (0.90–1.42)	.26
*P* for trend		<.001		.01		.09

Model 1: no adjustment was made for any covariates.

Model 2: adjusted for age, sex, race,education,marital status, PIR, BMI.

Model 3: adjusted for the covariates in model 2 plus hypertension, DM, hyperlipidemia, CVD, anemia, HbA1c, HDL-C, WBC, drinking status, physical activity, floss use.

BMI = body mass index, CI = confidence interval, CVD = cardiovascular disease, DM = diabetes mellitus, HbA1c = glycosylated hemoglobin level, HDL-C = high-density lipoprotein cholesterol, OR = odds ratio, PIR = poverty income ratio, RAR = the red blood cell distribution width to albumin ratio, Ref. = reference, WBC = white blood cell.

## 4. Discussion

This nationally representative cross-sectional study shows that, in adjusted models, RAR was positively associated with periodontitis. This association persisted even after conducting a sensitivity analysis using multiple methods to impute missing values. Furthermore, subgroup analyses confirmed the robustness of the association across different demographic and clinical factors.

Numerous previous studies have examined the association of RAR, a composite blood parameter, with a variety of diseases. Hao et al^[13]^ demonstrated that higher levels of RAR were associated with an increased risk of all-cause mortality in the general population (HR = 1.83; 95% CI: 1.76–1.90), and Wu et al^[16]^ demonstrated that higher levels of RAR were associated with an increased prevalence of renal stones in the general adult population (HR = 1.31; 95% CI: 1.16–1.47). In addition, Liu et al^[27]^found that RAR was positively associated with the prevalence of Parkinson disease (HR = 1.47; 95% CI: 1.16–1.86). However, the relationship between RAR and periodontitis remains unclear. In our study, after adjusting for age, sex, race, education, marital status, PIR, BMI, hypertension, DM, hyperlipidemia, CVD, anemia, HbA1c, HDL-C, WBC, drinking status, physical activity, and floss use, RAR remained associated with periodontitis (OR = 1.23; 95% CI: 1.02–1.49). When sensitivity analysis was conducted after multiple imputation of missing values, an association between RAR and periodontitis still persisted (OR = 1.14; 95% CI: 0.95–1.37).

In recent years, research on periodontitis has expanded significantly beyond the local oral environment, with increasing attention being paid to its association with systemic diseases.^[28,29]^ Elevated RDW reflects increased heterogeneity of erythrocyte volume, which is closely related to the inflammatory state. Sridharan et al^[9]^ found a significant positive correlation between the coefficient of variation in RDW and the surface area of periodontal inflammation, and identified a dose-response relationship between local periodontal inflammatory burden and changes in red blood cell morphology. In our study, we also found that the RDW in the moderately/ severe periodontitis group was significantly higher than that in the non/mild periodontitis group (13.22% vs 13.06%, *P < *.001). Pro-inflammatory cytokines (such as IL-6, TNF-α) in localized gingival tissues of patients with periodontitis can activate systemic inflammatory responses through the blood circulation.^[9]^ These cytokines may directly inhibit the maturation of bone marrow erythroid progenitor cells, leading to ineffective erythropoiesis and increased heterogeneity of erythrocyte volume, which is manifested as elevated RDW.^[13]^ Furthermore, it has been demonstrated that periodontitis interferes with iron metabolism by upregulating hepcidin levels, leading to chronic disease-associated anemia, which is manifested as an increased RDW and impaired hemoglobin synthesis.^[30]^

On the other hand, the ALB level is an important factor for assessing nutritional status and the severity of chronic diseases. There is a growing body of evidence showing that ALB levels in patients with periodontitis, especially those with severe or extensive forms, tend to be significantly lower than those in periodontally healthy individuals.^[20,31-33]^ A cohort study by Iwasaki et al^[33]^ further confirmed that lower ALB levels are associated with an accelerated rate of progression of CAL. In our study, the mean ALB level was significantly lower in the moderate/severe periodontitis group than in the non/mild periodontitis group (4.24 vs 4.30 g/dL, *P* < .001). Hypoalbuminemia is commonly observed in chronic inflammatory conditions and may result from reduced hepatic synthesis mediated by inflammatory factors, fluid leakage due to increased vascular permeability, and enhanced metabolism. Patients with periodontitis, especially those with severe disease, are often associated with decreased masticatory function, loss of appetite, or inadequate nutritional intake, which may further exacerbate the hypoalbuminemia state.^[34]^

As a composite parameter that reflects both elevated RDW and reduced ALB, RAR may provide a more comprehensive indication of the association with periodontitis than a single indicator. There may be a bidirectional association between RAR and periodontitis. Chronic local inflammation in the periodontium can inhibit albumin synthesis through an IL-6-mediated negative acute-phase response in hepatocytes. At the same time, the inflammatory microenvironment disrupts the maturation of erythroid progenitor cells, leading to an increase in RDW, ultimately, an elevation in RAR.^[35]^ Conversely, elevated RAR levels indicate that the body is in a state of high oxidative stress and low-protein nutrition, which can promote the release of pro-inflammatory factors such as TNF-α and IL-1β, potentially further exacerbating periodontal soft tissue destruction.^[20]^ At the same time, the immunosuppression caused by low ALB levels weakens the ability to clear local pathogens and may also accelerate the loss of periodontal attachment and alveolar bone resorption.

Furthermore, we implemented a stratified analysis to assess the potential impact of other variables on the results. Our findings revealed that age likely exerts a significant influence on the association between RAR and periodontitis. A key finding derived from the subgroup analysis when age was considered as a confounding factor was that RAR levels were significantly positively associated with periodontitis in individuals younger than 60 years, indicating that higher RAR in this age group is associated with an increased odds of periodontitis. This observation suggests that age may be a key factor influencing this association. The manifestation of this association is subject to critical determinants. In contrast, no significant association was observed between RAR levels and the prevalence of periodontitis in individuals older than 60 years. Notably, the absence of this association in the elderly population suggests that factors other than RAR may play a more dominant role in the odds of periodontitis among individuals in this age group. Overall, these findings underscore the importance of considering age as a key variable when examining the relationship between RAR and periodontitis, suggesting that the association between RAR and periodontitis may vary across different age groups.

However, there are several limitations to this study. First, the inherent limitations of the cross-sectional design prevented us from determining the direction of causality between RAR and periodontitis. Although the use of logistic regression analysis is in line with the norms of previous studies and the robustness of the findings was confirmed in the sensitivity analysis, additional longitudinal studies are needed to elucidate the association. Second, despite the inclusion of multiple confounders from previous studies and clinical experience, and the reduction of potential bias through subgroup analysis, residual confounders may still affect the final results. Third, the study population sample was drawn from the US population, limiting the overall representation of the general population.

## 5. Conclusions

This study suggests that a higher RAR level was associated with higher odds of periodontitis. Future research will focus on longitudinal research to validate our findings and explore the underlying mechanisms behind this association.

## Acknowledgments

The authors sincerely thank the National Health and Nutrition Examination Survey (NHANES) database for providing data. We thank Dr Jie Liu and Dr Muyang Liu (Chinese PLA General Hospital & Physician-Scientist Center of China) for their contribution to the statistical support, study design consultations and comments regarding the manuscript.

## Author contributions

**Conceptualization:** Lina Zhu, Ke Yang.

**Data curation:** Lina Zhu, Ke Yang.

**Formal analysis:** Lina Zhu.

**Funding acquisition:** Lina Zhu.

**Methodology:** Lina Zhu.

**Project administration:** Ke Yang.

**Resources:** Ke Yang.

**Software:** Lina Zhu, Ke Yang.

**Supervision:** Wenhao Qian.

**Validation:** Lina Zhu.

**Visualization:** Lina Zhu, Wenhao Qian.

**Writing – original draft:** Lina Zhu, Qian Zheng.

**Writing – review & editing:** Lina Zhu, Ke Yang.



## References

[1] Hajishengallis G. Interconnection of periodontal disease and comorbidities: evidence, mechanisms, and implications. Periodontol 2000. 2022;89:9–18. 35244969 10.1111/prd.12430 PMC9018559

[2] Chen MX Zhong YJ Dong QQ Wong HM Wen YF. Global, regional, and national burden of severe periodontitis, 1990-2019: an analysis of the Global Burden of Disease Study 2019. J Clin Periodontol. 2021;48:1165–88. 34101223 10.1111/jcpe.13506

[3] Alvarez C Abdalla H Sulliman S. RvE1 impacts the gingival inflammatory infiltrate by inhibiting the T cell response in experimental periodontitis. Front Immunol. 2021;12:664756. 34012448 10.3389/fimmu.2021.664756 PMC8126725

[4] Hajishengallis G Chavakis T. Local and systemic mechanisms linking periodontal disease and inflammatory comorbidities. Nat Rev Immunol. 2021;21:426–40. 33510490 10.1038/s41577-020-00488-6 PMC7841384

[5] Martínez-García M Hernández-Lemus E. Periodontal inflammation and systemic diseases: an overview. Front Physiol. 2021;12:709438. 34776994 10.3389/fphys.2021.709438 PMC8578868

[6] Liu Y Sun S Liu L. Association between the red blood cell distribution width-albumin ratio and cardiovascular diseases. Front Cardiovasc Med. 2025;12:1529533. 40271132 10.3389/fcvm.2025.1529533 PMC12014755

[7] Ainiwaer A Kadier K Abulizi A. Association of red cell distribution width (RDW) and the RDW to platelet count ratio with cardiovascular disease among US adults: a cross-sectional study based on the National Health and Nutrition Examination Survey 1999-2020. BMJ Open. 2023;13:e068148. 10.1136/bmjopen-2022-068148 PMC10016283 36914191

[8] Bhattacharya HS Srivastava R Gummaluri SS Agarwal MC Bhattacharya P Astekar MS. Comparison of blood parameters between periodontitis patients and healthy participants: a cross-sectional hematological study. J Oral Maxillofac Pathol. 2022;26:77–81. 35571313 10.4103/jomfp.jomfp_349_21 PMC9106243

[9] Sridharan S Sravani P Rao RJ. Coefficient of variation of red cell distribution width has correlations to periodontal inflamed surface area in non-obese hypertensive patients. J Int Acad Periodontol. 2021;23:106–14. 33929811

[10] Buranasin P Kominato H Mizutani K. Influence of reactive oxygen species on wound healing and tissue regeneration in periodontal and peri-implant tissues in diabetic patients. Antioxidants (Basel). 2023;12:1787. 37760090 10.3390/antiox12091787 PMC10525304

[11] Artigas A Wernerman J Arroyo V Vincent J-L Levy M. Role of albumin in diseases associated with severe systemic inflammation: pathophysiologic and clinical evidence in sepsis and in decompensated cirrhosis. J Crit Care. 2016;33:62–70. 26831575 10.1016/j.jcrc.2015.12.019

[12] Mousa N Salah M Elbaz S. Neutrophil percentage-to-albumin ratio is a new diagnostic marker for spontaneous bacterial peritonitis: a prospective multicenter study. Gut Pathog. 2024;16:18. 38561807 10.1186/s13099-024-00610-2 PMC10985869

[13] Hao M Jiang S Tang J. Ratio of red blood cell distribution width to albumin level and risk of mortality. JAMA Netw Open. 2024;7:e2413213. 38805227 10.1001/jamanetworkopen.2024.13213 PMC11134218

[14] Fu W Hu F Xu C. Association between red blood cell distribution width/albumin ratio and all-cause mortality or cardiovascular diseases mortality in patients with diabetic retinopathy: a cohort study. PLoS One. 2023;18:e0296019. 38128055 10.1371/journal.pone.0296019 PMC10735013

[15] Ma C Liang G Wang B. Clinical value of the red blood cell distribution width to albumin ratio in the assessment of prognosis in critically ill patients with sepsis: a retrospective analysis. J Thorac Dis. 2024;16:516–29. 38410549 10.21037/jtd-23-1696 PMC10894361

[16] Wu L Zhang Y Chen D. Association of red blood cell distribution width to albumin ratio with the prevalence of kidney stones among the general adult population. Immun Inflamm Dis. 2024;12:e70070. 39570091 10.1002/iid3.70070 PMC11580274

[17] Zipf G Chiappa M Porter KS Ostchega Y Lewis BG Dostal J. National Health and Nutrition Examination Survey: plan and operations, 1999-2010. Vital Health Stat 1. 2013;56:1–37. 25078429

[18] Eke PI Thornton-Evans GO Wei L Borgnakke WS Dye BA Genco RJ. Periodontitis in US adults: National Health and Nutrition Examination Survey 2009–2014. J Am Dent Assoc. 2018;149:576–88.e6. 29957185 10.1016/j.adaj.2018.04.023 PMC8094373

[19] Eke PI Page RC Wei L Thornton-Evans G Genco RJ. Update of the case definitions for population-based surveillance of periodontitis. J Periodontol. 2012;83:1449–54. 22420873 10.1902/jop.2012.110664 PMC6005373

[20] Xiang J Cai Y Yu Q Zhu Z Wang Y Chen R. Association between serum albumin and periodontitis across disease subgroups: a cross-sectional study. Int Dent J. 2025;75:100808. 40311190 10.1016/j.identj.2025.03.017 PMC12084511

[21] Xu J Jia Y Mao Z Wei X Qiu T Hu M. Association between serum uric acid, hyperuricemia and periodontitis: a cross-sectional study using NHANES data. BMC Oral Health. 2023;23:610. 37644435 10.1186/s12903-023-03320-4 PMC10466695

[22] Liu Y Wu Y Shi X. Association between blood lead and periodontitis among American adults: a cross-sectional study of the national health and nutrition examination survey. Front Pharmacol. 2024;15:1420613. 39619608 10.3389/fphar.2024.1420613 PMC11604452

[23] Hu S Guo Q Wang S. Supplementation of serum albumin is associated with improved pulmonary function: NHANES 2013-2014. Front Physiol. 2022;13:948370. 36262258 10.3389/fphys.2022.948370 PMC9574070

[24] Chen J Li Y Chen X. The albumin-globulin ratio is associated with periodontitis in American adults: results from the NHANES 2009-2014. Sci Rep. 2025;15:27626. 40730566 10.1038/s41598-025-06416-9 PMC12307635

[25] Zheng Z Xie X Wang L. Association between neutrophil-percentage-to-albumin ratio and periodontitis: insights from a population-based study. Front Nutr. 2025;12:1551349. 40290655 10.3389/fnut.2025.1551349 PMC12021642

[26] Kimura H Tanaka K Saito H. Impact of red blood cell distribution width-albumin ratio on prognosis of patients with CKD. Sci Rep. 2023;13:15774. 37737253 10.1038/s41598-023-42986-2 PMC10516924

[27] Liu F Ran Q Li Z Chen J. The red blood cell distribution width-to-albumin ratio’s role in Parkinson’s disease: a cross-sectional study. J Clin Med. 2025;14:4908. 40725602 10.3390/jcm14144908 PMC12296135

[28] Walther K-A Gröger S Vogler JAH Wöstmann B Meyle J. Inflammation indices in association with periodontitis and cancer. Periodontol 2000. 2024;96:281–315. 39317462 10.1111/prd.12612 PMC11579835

[29] Tran AH Zaidi AH Bolger AF.; American Heart Association Cardiovascular Disease Prevention Committee of the Council on Lifelong Congenital Heart Disease and Heart Health in the Young; Council on Clinical Cardiology; Stroke Council; Council on Basic Cardiovascular Sciences; and Council on Cardiovascular and Stroke Nursing. Periodontal disease and atherosclerotic cardiovascular disease: a scientific statement from the American Heart Association. Circulation. 2026;153:e73–88. 41399933 10.1161/CIR.0000000000001390

[30] Han Y Luo Z Yue ZG. The tendency of anemia of inflammation in periodontal diseases. Clin Sci (Lond). 2023;137:251–64. 36705427 10.1042/CS20220524 PMC9908573

[31] Kaur N Kaur N Sarangal V. A study to evaluate the correlation of serum albumin levels with chronic periodontitis. Indian J Dent Res. 2015;26:11–4. 25961608 10.4103/0970-9290.156788

[32] Naghsh N Sabet NK Vahidi F Mogharehabed A Yaghini J. Relationship between periodontal disease and serum factors in patients undergoing hemodialysis. Open Dent J. 2017;11:701–9. 29399214 10.2174/1874210601711010701 PMC5759098

[33] Iwasaki M Yoshihara A Hirotomi T Ogawa H Hanada N Miyazaki H. Longitudinal study on the relationship between serum albumin and periodontal disease. J Clin Periodontol. 2008;35:291–6. 18353078 10.1111/j.1600-051X.2008.01215.x

[34] Isola G. The impact of diet, nutrition and nutraceuticals on oral and periodontal health. Nutrients. 2020;12:2724. 32899964 10.3390/nu12092724 PMC7551041

[35] Lei R Liang H Ding X. Red cell distribution width to albumin ratio (RAR) as a potential biomarker of coronary heart disease (CHD): insights from a cross-sectional study. Medicine (Baltimore). 2025;104:e46663. 41432369 10.1097/MD.0000000000046663 PMC12727370

