# Supplementary material for: Association between red blood cell distribution width to albumin ratio and periodontitis in US adults: A cross-sectional study
Source: Medicine (Baltimore). 2026 Aug 28;105(35):e50414. doi: 10.1097/MD.0000000000050414 (PMC13529138; doi:10.1097/MD.0000000000050414)
Supplement: Supplementary file 1 [file medi-105-e50414-s001.docx]

# **Association between red blood cell distribution width to albumin ratio and periodontitis in US adults** : a cross-sectional study

**Supplementary marterials**

**Table S1.** Weighted univariate regression analysis

**Table S1.** Weighted univariate regression analysis

| **Variable** | **OR (95%CI)** | ***P-*value** |
| --- | --- | --- |
| **Age (**years) | 1.04 (1.04-1.05) | <.001 |
| **Sex**:Female vs Male | 0.51 (0.47-0.55) | <.001 |
| **Race**:ref.=Non-Hispanic White |  |  |
| Non-Hispanic Black | 2.05 (1.82-2.29) | <.001 |
| Mexican American | 2.07 (1.81-2.36) | <.001 |
| Others | 1.26 (1.12-1.41) | <.001 |
| **Marital Status**:ref.= Married/Living with partner |  |  |
| Never married/Other | 1.32 (1.21-1.45) | <.001 |
| **PIR**:ref.=PIR≤1.30 |  |  |
| 1.31-3.50 | 0.68 (0.61-0.76) | <.001 |
| ＞3.50 | 0.33 (0.30-0.37) | <.001 |
| **Education**:ref.=Less than 9th Grade |  |  |
| High School or Equivalent | 0.60 (0.50-0.72) | <.001 |
| Above high School | 0.25 (0.21-0.29) | <.001 |
| **Smoking**:ref.=Never |  |  |
| Former | 1.76 (1.59-1.95) | <.001 |
| Now | 2.76 (2.45-3.10) | <.001 |
| **Drinking**:ref.=Never |  |  |
| Former | 1.66 (1.42-1.94) | <.001 |
| Current | 0.80 (0.72-0.88) | <.001 |
| **BMI** | 1.00 (1.00-1.01) | .17 |
| **Hypertension**:ref.=No |  |  |
| Yes | 1.90 (1.74-2.07) | <.001 |
| **DM**: ref.= No |  |  |
| Yes | 2.17 (1.94-2.42) | <.001 |
| **Hyperlipidemia**:ref.=No |  |  |
| Yes | 1.21 (1.10-1.34) | <.001 |
| **CVD**: ref.= No |  |  |
| Yes | 2.44 (2.09-2.86) | <.001 |
| **Anemia**: ref.= No |  |  |
| Yes | 1.38 (1.19-1.59) | <.001 |
| **Floss use**:ref.= Never |  |  |
| Rarely | 0.49 (0.43-0.56) | <.001 |
| Moderately | 0.42 (0.37-0.48) | <.001 |
| Frequently | 0.61 (0.55-0.68) | <.001 |
| **Physical activity**: ref.= Inactive |  |  |
| Insufficiently active | 0.70 (0.60-0.80) | <.001 |
| Active | 0.72 (0.65-0.80) | <.001 |
| **HbA1c** | 1.43 (1.36-1.50) | <.001 |
| **HDL-C** | 0.76 (0.68-0.84) | <.001 |
| **WBC** | 1.07 (1.05-1.09) | <.001 |

Abbreviations: PIR, poverty income ratio; BMI, Body Mass Index; DM, Diabetes Mellitus; CVD, Cardiovascular Disease; HbA1c, glycosylated haemoglobin level; HDL-C, high-Density Lipoprotein Cholesterol; WBC, white blood cell
